# The sialate *O*-acetylesterase EstA from gut *Bacteroidetes* species enables sialidase-mediated cross-species foraging of 9-*O*-acetylated sialoglycans

**DOI:** 10.1074/jbc.M116.769232

**Published:** 2017-05-19

**Authors:** Lloyd S. Robinson, Warren G. Lewis, Amanda L. Lewis

**Affiliations:** From the Departments of ‡ Molecular Microbiology and; § Medicine, Center for Women's Infectious Disease Research, Washington University School of Medicine, St. Louis, Missouri 63110

**Keywords:** Escherichia coli (E. coli), microbiome, mucin, mucus, sialic acid, sialidase, Bacteroides fragilis, Bacteroides thetaiototaomicron, O-acetyl esterase, O-acetylation

## Abstract

The gut harbors many symbiotic, commensal, and pathogenic microbes that break down and metabolize host carbohydrates. Sialic acids are prominent outermost carbohydrates on host glycoproteins called mucins and protect underlying glycan chains from enzymatic degradation. Sialidases produced by some members of the colonic microbiota can promote the expansion of several potential pathogens (*e.g. Clostridium difficile*, *Salmonella*, and *Escherichia coli*) that do not produce sialidases. *O*-Acetyl ester modifications of sialic acids help resist the action of many sialidases and are present at high levels in the mammalian colon. However, some gut bacteria, in turn, produce sialylate-*O*-acetylesterases to remove them. Here, we investigated *O*-acetyl ester removal and sialic acid degradation by *Bacteroidetes* sialate-*O*-acetylesterases and sialidases, respectively, and subsequent utilization of host sialic acids by both commensal and pathogenic *E. coli* strains. *In vitro* foraging studies demonstrated that sialidase-dependent *E. coli* growth on mucin is enabled by *Bacteroides* EstA, a sialate *O*-acetylesterase acting on glycosidically linked sialylate-*O*-acetylesterase substrates, particularly at neutral pH. Biochemical studies suggested that spontaneous migration of *O*-acetyl esters on the sialic acid side chain, which can occur at colonic pH, may serve as a switch controlling EstA-assisted sialic acid liberation. Specifically, EstA did not act on *O*-acetyl esters in their initial 7-position. However, following migration to the 9-position, glycans with *O*-acetyl esters became susceptible to the sequential actions of bacterial esterases and sialidases. We conclude that EstA specifically unlocks the nutritive potential of 9-*O*-acetylated mucus sialic acids for foraging by bacteria that otherwise are prevented from accessing this carbon source.

## Introduction

The equilibrium between gut microbes and secreted mucus is vital to maintaining intestinal health (1–3). Heavily glycosylated mucus components are produced in abundance by specialized cells in the mammalian intestinal mucosa and act as a physical and biochemical barrier to protect the mammalian host from inappropriate interactions at the host-microbe interface in the distal colon and rectum (4–7). Components of the outermost “loose” mucus layer are an important source of nutrition for symbiotic bacteria, which have evolved highly efficient means to digest the carbohydrate chains therein (2, 8). These carbohydrate chains are often capped with unique 9-carbon-backbone monosaccharides called sialic acids, which protect sialoglycoproteins from the action of exoglycosidases, some endoglycosidases, and certain proteases (9–11). Sialic acids come in many forms, the most common of which is *N*-acetylneuraminic acid (Neu5Ac)^3^ (12). A number of intestinal bacteria (both symbionts and pathogens) express sialidases (also known as neuraminidases), allowing these bacteria to remove sialic acids from sialoglycans (13). Bacterial sialidases have been linked with many strategies and mechanisms for survival or proliferation in the host, including the utilization of sialic acids or underlying carbohydrates as carbon sources, interaction/adhesion to underlying carbohydrates, immunomodulatory activities, or to break down mucus, allowing better access to the epithelial surface underneath.

The modification of sialic acids with *O*-acetyl esters has long been implicated as a host adaptation that protects the integrity of the mucus barrier because of the relative resistance of *O*-acetylated sialic acids against sialidases (14–19). However, studies have indicated that cell-free supernatants of human and rodent fecal extracts also contain *O*-acetyl esterases capable of acting on *O*-acetylated sialic acids within glycosidically linked sialoglycan substrates (17, 20, 21). In fact, similar to sialidases, sialic acid *O*-acetyl esterases are widely distributed among human intestinal bacteria and are often expressed on the bacterial cell surface or secreted into culture medium (18). More recent studies in members of the oral *Bacteroidetes* further implicated *O*-acetyl esterases in glycan foraging within in the oral mucosa and demonstrated the genetic basis of the enzyme (22). These examples stand in contrast to other intracellular bacterial sialic acid *O*-acetyl esterases that have been shown to participate in the biosynthesis or catabolism of sialic acids by acting on free or nucleotide-linked sialic acid esters rather than complex glycosidically linked substrates (23–25). Finally, we note that despite the constitutive presence of sialidases and sialic acid *O*-acetyl esterases in the large intestine, glycan chains containing sialic acids and their *O*-acetylated counterparts are highly abundant in the distal colon and rectum (26–29). These findings suggest that in normal healthy individuals, the rate of production of mucin sialoglycan chains exceeds the rate of bacterial digestion or that some mucin sialoglycans remain resistant to the action of bacterial enzymes.

The delicate balance of species within gut bacterial communities relies on a web of metabolites produced by individuals or sectors of the population and consumed by other subsets within the community. In regards to complex sugars or glycans, it has been shown that one sector of intestinal bacteria within a population can secrete enzymes that digest these molecules, whereas another set of organisms can take advantage of this situation by scavenging the liberated sugars, referred to previously as “public goods” (30). Other recent studies show that this concept applies to mucus sialic acids and can facilitate the outgrowth of pathogens in the gut (31, 32). For example, one recent study showed that antibiotic treatment kills sialidase-producing members of the microbiota in the mouse intestine, resulting in an imbalance in bacterial enzymes and metabolic pathways, leading to a transient spike in liberated sialic acids. This in turn supports the metabolism and outgrowth of the antibiotic-associated pathogens *Clostridium difficile* and *Salmonella*, which do not encode sialidases of their own (31). Another recent study showed that the expansion of certain *Bacteroides* species during dextran sulfate sodium (DSS)-induced colitis (a model that resembles ulcerative colitis) led to similarly increased levels of sialidase and subsequent outgrowth of *Escherichia coli*, which was dependent on the ability of *E. coli* to catabolize sialic acid (32) and further exacerbated the colitis. There are in fact many examples of such bacteria that do not encode sialidases but have functional (or predicted) sialic acid catabolic pathways. Another example of sialic acid cross-feeding involves the genus *Bifidobacteria*, in which most strains encode the machinery to catabolize free sialic acid, but a small subset encodes their own endogenous sialidases (33–38). In short, the cross-species feeding of host sialic acids between members of the gut microbiome is likely to have broad significance for our understanding of the microbiota and its role in health and disease.

Based on the very high level of sialic acid *O*-acetylation in the distal colon and rectum (26, 29), we hypothesized that the use of sialic acids as public goods in this niche likely relies on the action of sialic acid *O*-acetyl esterases. Here we use *in vitro* models to understand the interplay between host *O*-acetyl esters and bacterial *O*-acetylesterases and sialidases, and the conditions under which these enzymes work together to liberate sialic acids for use by microbes encoding sialic acid catabolic machinery. Specifically, we investigate 1) the role of sialic acid *O*-acetylation as an impediment to the liberation and utilization of sialic acids by intestinal bacteria, 2) the role of bacterial O-acetyl esterase in removing *O*-acetyl esters of mucin sialic acids to stimulate cross-species utilization of sialic acids, and 3) the role of *O*-acetyl placement and migration in the susceptibility of sialic acid *O*-acetyl esters to digestion by O-acetyl esterase.

## Results

Members of the genus *Bacteroides* are an important contributor of bacterial sialidases (13, 18, 31, 39, 40) and sialic acid *O*-acetyl esterases (18, 39) relevant to the colonic niche. Thus, sialidases from *Bacteroides fragilis* (WP_010992682) and*Bacteroides thetaiotaomicron* (WP_008766031) were cloned, expressed in *E. coli*, and purified for use in our *in vitro* models of cooperative metabolism. Notably, whereas *B. fragilis* has been previously shown to utilize its sialidase in the acquisition of sialic acid, *B. thetaiotaomicron* does not have a sialic acid catabolic gene cluster and does not utilize the sialic acid it liberates (41). In contrast, *E. coli* encodes a pathway for the transport and catabolism of sialic acid and has been shown to utilize sialic acid as a carbon source (42). Although some strains of *E. coli* have been noted to encode open reading frames annotated as sialidases (13, 43–45), these are bacteriophage tailspike endosialidases, which degrade the α2–8-linked polysialic acid capsule of *E. coli* K1 strains (46). As yet, there have been no functionally recognized sialidases in *E. coli* that act on host sialic acids that are normally found in mucosal contexts.

**Figure 1.**
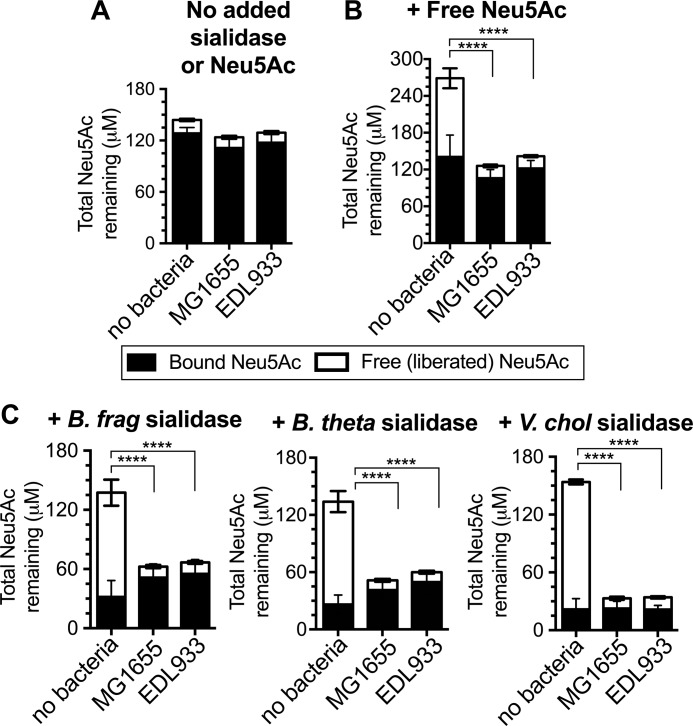
***E. coli* is ill-equipped to catabolize sialic acids in the bound form but is enabled by accessory sialidases expressed by other intestinal bacteria.** Concentration of sialic acid (*Neu5Ac*) remaining in LB following overnight culture of *E. coli* (commensal strain MG1655 and enterohemorrhagic strain EDL933). The figure key showing bound (*black bars*) and free sialic acid (*white bars*) applies to all panels. *E. coli* was cultured with no added sialidase or sialic acid (*A*), 100 μm free Neu5Ac added to media (*B*), or purified His-tagged *B. fragilis* or *B. thetaiotaomicron* sialidase added to media (*C*) prior to *E. coli* inoculation. Similar results were obtained in Columbia media. Another common sialic acid, Neu5Gc, is also liberated by gut sialidases and consumed by *E. coli* in this setting. Statistical comparisons (one-way ANOVA followed by pairwise *t* tests between the uninoculated control and each experimental condition, adjusted for multiple comparisons by the Bonferroni method) were performed using the free sialic acid values. ****, *p* < 0.0001 The data shown are combined from three to five independent experiments, each of which was performed with one biological replicate and one technical replicate.

To directly examine the ability of *E. coli* (MG1655 (a commensal strain) and EDL933 (an enterohemorrhagic strain)) to access sialic acids within glycosidically bound sialoglycan substrates, sialic acid levels in cell free supernatants were measured after overnight growth in lysogeny broth (LB). Briefly, sialic acids can be measured using a fluorescent derivatization procedure that only works on sialic acids in the free (liberated) monosaccharide form. Thus, we can measure levels of free and total sialic acids (47) (and by extension, bound sialic acids) in LB by performing the derivatization with or without prior mild acid hydrolysis to liberate sialic acids. During routine growth in culture media, sialic acids were mostly found in the bound state within glycans. These sialic acids within sialoglycans were *not* accessible to *E. coli* (Fig. 1*A*). In contrast, both strains of *E. coli* readily consumed free sialic acid that was added exogenously to the medium (Fig. 1*B*). Similarly, the addition of purified sialidases from intestinal bacteria converted bound sialic acids into “public goods” that could be metabolized by *E. coli* (Fig. 1*C*). We used well described sialidases produced by symbionts, commensals, and pathogens, including 1) the prevalent gut symbiont *B. thetaiotaomicron* (40), 2) the common human commensal and opportunistic pathogen *B. fragilis* (48), and 3) the water-borne bacterial intestinal pathogen *Vibrio cholerae* (49). The results of these experiments illustrate that sialidases known to be present within the context of the healthy gut and during infection can enable the use of sialic acids by other members of the community. These experiments also demonstrate the suitability of *E. coli* as a model organism for investigating sialic acid scavenging behaviors; it neither expresses sialidase activity (as shown by the lack of release in Fig. 1*A*) nor is capable of accessing sialic acids in bound form without the help of an accessory sialidase.

**Figure 2.**
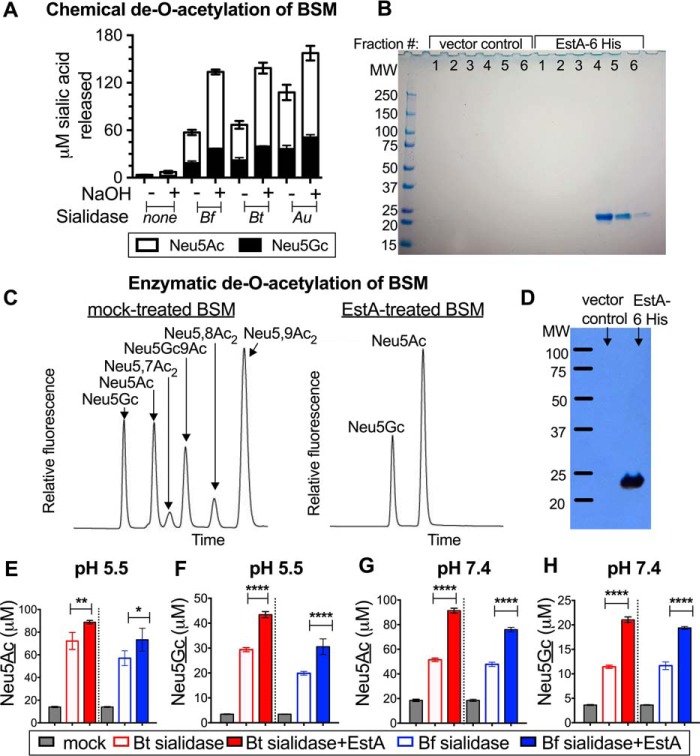
**The sialic acid *O*-acetyl esterase EstA augments liberation of mucin sialic acids by sialidases of gut bacteria.** BSM treated with and without mild base was incubated with *B. thetaiotaomicron* (*Bt*), *B. fragilis* (*Bf*), and *A. ureafaciens* (*Au*) sialidases. *A*, base treatment enhances sialic acid release by sialidases, as quantified by DMB HPLC. *B*, expression and purification of *B. fragilis* EstA-His_6_. Nickel-purified fractions derived from *E. coli* expressing EstA-His_6_ or vector alone were resolved by SDS-PAGE and stained with Coomassie Blue. *C*, extended treatment of mucin with *B. fragilis* esterase results in removal of acetyl groups, as shown by HPLC traces of DMB-derivatized sialic acids treated with EstA compared with mock-treated control (parallel nickel chromatography of *E. coli* periplasm from empty vector-containing *E. coli*). *D*, Western blot analysis with monoclonal anti-His_6_ antibody on fraction 5 of purified EstA-His_6_ or fraction 5 of the vector control. *E–H*, EstA augments sialic acid release from mammalian mucin by *B. fragilis* (*Bf*, *blue*) and *B. thetaiotaomicron* (*Bt*, *red*) NanH sialidases. Enzyme(s) were incubated with BSM at pH 5.5 (*E* and *F*) and pH 7.4 (*G* and *H*). Liberated sialic acids were base-treated prior to DMB-HPLC measurement. Neu5Ac (*E* and *G*) and Neu5Gc (*F* and *H*) release by each sialidase were measured under each condition. Statistical significance was examined using one-way ANOVA followed by pairwise *t* tests adjusted for multiple comparisons by the Bonferroni method. *, *p* < 0.05; **, *p* < 0.01; ****, *p* < 0.0001. The experiment presented in *A* was performed three times with one biological replicate and one technical replicate. The experiment shown in *C* was done four times with two biological replicates and one technical replicate each. The experiment shown in *E–H* was performed once with one biological replicate and three technical replicates. Several iterations were performed at only one pH with similar results.

To establish the influence of sialic acid *O*-acetylation on mobilization of sialic acids from mucin substrates by sialidase-producing bacteria (*B. thetaiotaomicron* and *B. fragilis*), we used bovine submaxillary mucin (BSM), a “virgin” preparation of mammalian mucin that is purified directly from the gland, sparing the mucin from exposure to hydrolases of the microbiota. As such, freshly prepared BSM consists of several percent sialic acid by weight and contains a high level of sialic acid *O*-acetylation. First, BSM was either treated with mild sodium hydroxide to remove *O*-acetyl esters or was mock-treated in parallel prior to sialidase exposure as described under “Experimental procedures.” Fluorescent derivatization of free sialic acids from the control reactions demonstrated that there was a very low level of free sialic acid present in samples that were *not* exposed to sialidase (Fig. 2*A*). However, in samples exposed to sialidases, *O*-acetylated (mock-treated) mucin sialic acids were considerably more resistant to the action of sialidases than NaOH-treated (de-*O*-acetylated) mucin sialic acids. Thus, consistent with previous studies using different approaches (17, 18, 20), our data show that chemical removal of mucin *O*-acetyl esters augments the mobilization of sialic acids by sialidases of gut bacteria. As previously reported, the sialidase from *Arthrobacter ureafaciens* is less sensitive to *O*-acetylation and is capable of releasing more sialic acid from the mock-treated (*O*-acetylated) BSM compared with the *Bacteroides* sialidases (Fig. 2*A*).

The first evidence for the putative genetic basis of *B. fragilis* sialic acid *O*-acetyl esterase activity showed that the enzyme EstA was capable of producing acetate after incubation with mucin or with pNP-acetate (39). Next, we directly investigated the ability of recombinant purified EstA to remove *O*-acetyl esters from mucin sialic acids and specifically the *O*-acetyl esters of the sialic acid side chain (carbon positions 7 and 9), resulting in the corresponding non-*O*-acetylated sialic acid product. Briefly, EstA was expressed as a 6-histidine-tagged protein in *E. coli* and isolated from the periplasmic fraction with nickel affinity resin as described under “Experimental Procedures.” Expression and purification of EstA-His_6_ were verified by SDS-PAGE followed by staining with Coomassie Blue and by Western blotting with anti-His_6_ monoclonal antibody (Fig. 2, *B* and *D*). BSM was incubated in the presence or absence of EstA, and product formation was monitored after a 2-h incubation at 37 °C by HPLC of sialic acids released by treatment with *A. ureafaciens* sialidase (AUS). In the EstA-treated samples, peaks corresponding to *O*-acetylated sialic acids disappeared, whereas several species of *O*-acetylated sialic acids remained in the mock-treated samples (Fig. 2*C*). Thus, EstA is capable of removing *O*-acetyl esters from the sialic acid side chain of *O*-linked mucin glycans.

Sialic acid *O*-acetyl esterase activity has been previously described in cell free fecal extracts and is expressed by gut bacterial isolates in both secreted and cell surface forms (17, 18, 20). To determine whether EstA-mediated removal of *O*-acetyl esters from mucin sialic acids enables their release by sialidases of gut bacteria, we treated BSM with sialidases from *B. fragilis* and *B. thetaiotaomicron* in the presence or absence of EstA and monitored liberation of sialic acid into the free form. These reactions also modeled the effects of pH on sialic acid liberation by gut sialidases using conditions that represent the upper and lower limits of pH in the large intestine (50, 51). As described under “Experimental procedures,” overall sialic acid release was followed by base treatment of the samples to collapse all the sialic acids into two peaks, Neu5Ac and *N*-glycolylneuraminic acid (Neu5Gc). This facilitated the accurate quantitation of sialic acids, which can only be detected in our derivatization reaction if first liberated. Consistent with previous findings that many sialidases have optimal activity at somewhat acidic pH, we found that *Bacteroides* sialidases liberated significantly less sialic acid at pH 7.4, a pH more physiologically relevant for the rectum, than at pH 5.5, which is more relevant to the proximal colon (compare *open bars* between pH conditions in Fig. 2, *E–H*). Under both pH conditions, the addition of EstA to the reactions significantly improved the release of sialic acids by gut bacterial sialidases. This improvement was most striking at a pH physiologically relevant to the rectum (pH 7.4; Fig. 2, *E–H*). Specifically, at pH 5.5, the sialidases released 23–29% more Neu5Ac (Fig. 2*E*) and 48–53% more Neu5Gc (Fig. 2*F*) in the presence of EstA, whereas at pH 7.4 the sialidases released 59–78% more Neu5Ac (Fig. 2*G*) and 66–84% more Neu5Gc (Fig. 2*H*) in the presence of EstA. In conclusion, *B. fragilis* EstA hydrolyzes *O*-acetyl esters from mucin sialic acids in the glycosidically bound form and improves sialic acid liberation by gut sialidases at the physiologic pH of the colon.

**Figure 3.**
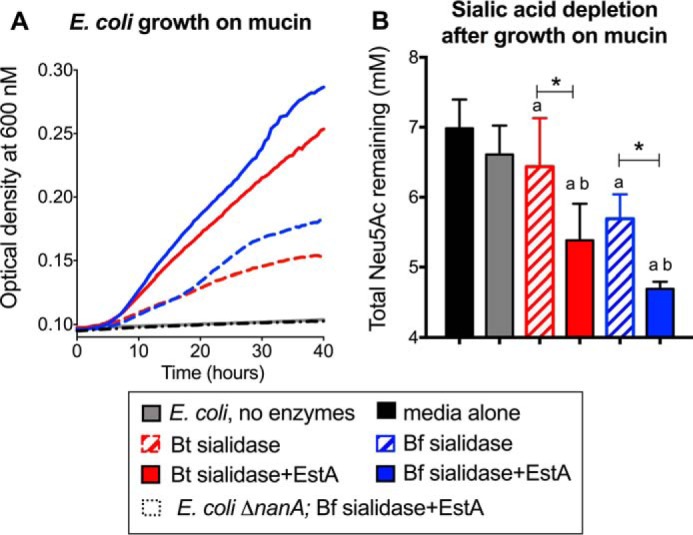
**EstA enhances the nutrative potential of mucin during community-driven foraging.** *E. coli* MG1655 was grown in M63 minimal medium supplemented with bovine submaxillary mucin in the presence of different enzyme combinations as described under “Experimental procedures.” *A*, growth was monitored by optical density (600 nm) readings taken every 2 min using a Tecan M200 plate reader. *B*, *E. coli* depletion of mucin sialic acids was examined by measuring total Neu5Ac levels in spent media at the end of the growth experiment. ANOVA with Dunnett's post test was used to examine statistical significance compared with medium alone and *E. coli* alone controls: different from media alone (*a*, *black bar*), *p* < 0.05; different from *E. coli*, no enzymes (*b*, *gray bars*). One-way ANOVA followed by pairwise *t* tests adjusted for multiple comparisons by the Bonferroni method was used to further evaluate differences between growth under conditions with sialidase alone *versus* sialidase + EstA. *, *p* < 0.05. This experiment was performed once with one biological replicate and one technical replicate and once with three biological replicates with one technical replicate each. It was also done as end-point assays in culture tubes, wherein only initial and final optical density measurements were taken. *Bt*, *red*, *B. thetaiotaomicron*; *Bf*, *blue*, *B. fragilis*.

Next, experiments were designed to investigate whether EstA promotes community foraging of mucin, leading to the expansion of sialic acid-scavenging bacteria. Here we evaluated the ability of *E. coli* to grow in minimal medium supplemented with mucin as its sole carbon source and the roles of sialidase and *O*-acetyl esterase in potentiating sialic acid-dependent growth. The negative control (*E. coli* without enzyme addition) confirms that the bacterium cannot utilize mucin or grow in the absence of enzymatic assistance from other community members. In contrast, the addition of either *B. thetaiotaomicron* or *B. fragilis* sialidase led to a significant expansion of *E. coli* (Fig. 3*A*, *dashed lines*). Consistent with the increased liberation of sialic acid in the presence of esterase (Fig. 2), the addition of EstA together with sialidase further encourages the proliferation of sialic acid scavenging bacteria (Fig. 3*A*). In contrast, an isogenic *E. coli* strain lacking sialic acid lyase (Δ*nanA*) was unable to grow on mucin under these conditions, even in the presence of sialidase and *O*-acetylesterase (Fig. 3*A*, *dashed black line*). Studies of certain *Bifidobacterium* and *Bacteroides* species indicate that sialic acid liberation serves the primary function of unlocking underlying carbohydrates (38, 41, 52). The fact that the sialate lyase-deficient *E. coli* strain exhibited no growth in the presence of sialidase and esterase demonstrates that the bacterial growth observed in these experiments is solely due to the catabolism of sialic acids. Sialic acids were significantly depleted by *E. coli* compared with medium-alone controls when bacterial sialidases were present (Fig. 3*B*, *hatched bars*), supporting the interpretation that sialic acid foraging is occurring under these conditions. Consistent with the enhanced growth observed in experimental groups with both esterase and sialidase *versus* those with sialidase alone (Fig. 3*A*, *dashed versus solid lines*), EstA further enhanced the ability of *E. coli* to deplete sialic acids from the medium in the presence of added sialidase (Fig. 3*B*, *solid red* and *blue bars*). Finally, *E. coli* was recently shown to use its own intracellular *O*-acetyl esterase (NanS) to catabolize free 9-*O*-acetylated sialic acid (25). The boost in *E. coli* growth in the presence of *B. fragilis* EstA compared with sialidase alone strongly supports the conclusion that extracellular EstA rather than intracellular NanS is responsible for the sialate lyase-dependent, sialic acid-specific enhancement of growth.

In theory, the removal of sialate esters from either position 7 or 9 would result in Neu5Ac as the reaction product. However, a clear understanding of enzyme action at these different positions is confounded by the propensity of *O*-acetyl esters on the 3-carbon sialic acid side chain to migrate. Specifically, sialic acids *O*-acetylated at C-7 of sialic acid are prone to pH-dependent (essentially unidirectional) migration along the sialic acid side chain to C-9 (53–57). However, little is known about the biological significance of *O*-acetyl migration, in large part because of the experimental challenge posed by biological conditions and analytical methods, which often result in extensive migration and loss of *O*-acetyl esters (58, 59). Part of the difficulty in interpreting the exact site(s) of action of EstA using bovine submaxillary mucin as substrate is due to the complexity of analytical profiles of bovine mucin sialic acids (containing both Neu5Ac and Neu5Gc, each with multiple *O*-acetylated species on the side chain). The problem is compounded by the notorious lability and migration of *O*-acetylation on the sialic acid side chain.

We therefore investigated the role of sialic acid *O*-acetyl placement and migration on EstA activity using the simpler *O*-acetylated and sialylated capsular polysaccharide of the gut commensal bacterium group B *Streptococcus* (GBS) (60). The GBS capsular polysaccharide mimics mammalian glycan structures and contains *O*-acetylated sialic acids (57). The advantages of using GBS as an *O*-acetylesterase substrate are 5-fold: 1) it can be easily cultured to produce high levels of *O*-acetylation that is freshly added at the 7-carbon position; 2) it allows the study of glycosidically linked *O*-acetylated sialic acids without time-consuming substrate isolation steps; 3) it offers a simplified substrate with the major *O*-acetyl ester in the 7-position, which can be deliberately migrated to the 9-position for comparison (56); 4) following treatment with EstA, it can be easily pelleted and washed repeatedly, removing esterase and ensuring that sialic acids released by subsequent sialidase treatment for HPLC analysis could only have been deacetylated in the bound condition; and 5) the organism synthesizes only Neu5Ac, whereas mammalian mucins usually contain mixtures of Neu5Ac and Neu5Gc, each with several sites of *O*-acetylation, resulting in a complex pattern of HPLC peaks that can complicate experimental interpretation. In short, GBS serves as an excellent model system for investigating EstA activity on *O*-acetyl esters at C-7 and C-9. We used a strain of GBS with high levels of *O*-acetylation resulting from the active site mutation of an intracellular sialic acid *O*-acetyl esterase that GBS itself expresses (23). This strain was chosen for our experiments both because of its high levels of capsular sialic acid *O*-acetylation (up to 80% of sialic acids) and because its use eliminates any possibility that the action of the GBS esterase might hinder interpretation of the EstA activity assays.

**Figure 4.**
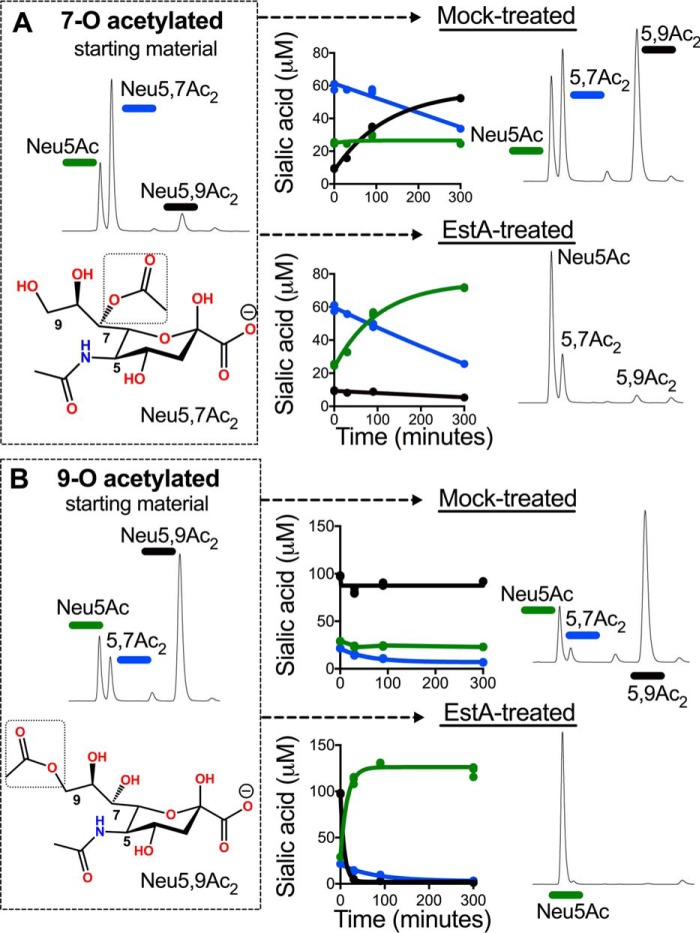
**EstA acts on 9-*O*- but not 7-*O*-acetyl esters of sialic acid.** GBS capsular polysaccharide mimics host glycans and can generate high levels of sialic acid O-acetyl esters that begin at the 7-carbon position and can be migrated to the 9-carbon position. We used the intact capsule on the surface of live GBS as a model system to define the specificity of EstA. GBS sialic acids that were maintained at the 7-carbon position or induced to migrate to the 9-carbon position as described under “Experimental procedures” were used as a substrate for purified EstA. After incubation of EstA with GBS bearing 7-*O*- (*A*) or 9-*O*-acetylated (*B*) sialic acids in PBS for 30, 90, and 300 min at 37C, bacteria were washed, and their sialic acids were released with *A. ureafaciens* sialidase and analyzed by DMB-HPLC. The HPLC traces of mock and EstA-treated samples are from the final, 300-min time point. This experiment was performed twice with one biological replicate and three technical replicates each.

To assess the activity of EstA on 7- and 9-*O*-acetyl esters, we combined EstA with preparations of GBS containing these glycosidically linked sialoglycan substrates. In these experiments, one portion of log phase GBS culture was resuspended in pH 9 Tris buffer and incubated for 30 min at 37 °C to migrate *O*-acetyl ester groups to the 9-carbon position. Another portion of culture was kept at neutral pH (in PBS) on ice in parallel, which retains *O*-acetyl groups at the 7-carbon position (see HPLC traces of the 7-*O*- and 9-*O*-acetylated starting material shown in Fig. 4). GBS cells were incubated with purified EstA-His or mock-treated as described under “Experimental procedures.” The assay was performed at a neutral pH relevant to the distal colon and rectum. The extent and placement of remaining *O*-acetyl esters was monitored at 0, 30, 90, and 300 min of incubation. Using 7-*O*-acetylated sialic acids as substrate, the EstA- and mock-treated samples experienced a similar net conversion of 7-*O*-acetyl esters (Fig. 4*A*). In the mock-treated sample, the progressive loss of *O*-acetylation at C-7 was accompanied by corresponding increases in the levels of 9-*O*-acetylated sialic acid indicative of *O*-acetyl migration at neutral pH. In contrast, EstA-treated samples exhibited losses of *O*-acetylation at C-7 that corresponded with slowly increasing levels of unmodified Neu5Ac. When we used 9-*O*-acetylated sialic acids as substrate, 9-*O*-acetyl esters were rapidly and completely hydrolyzed in the presence of EstA, whereas the mock-treated sample retained the 9-*O*-acetyl esters at high levels (Fig. 4*B*). These results strongly suggest that 7-*O*-acetylation is resistant to the action of the EstA O-acetylesterase, whereas 9-*O*-acetylation is highly sensitive.

## Discussion

The carbohydrates available to microbes in the gut can have a dramatic influence on the dynamic composition of the microbiota (2, 8, 61–64). In particular, recent studies show that cross-species feeding of host sialic acids, made available by the secreted sialidases of normal members of the microbiota, can facilitate intestinal infection and dysbiosis (31, 32). Here we demonstrate that *O*-acetylation of host sialic acid limits sialidase liberation of this carbon source for use by sialic acid scavengers. The *O*-acetylesterase EstA overrides this limitation, acting under neutral pH conditions relevant to the distal colon to improve the accessibility of sialic acids to gut bacterial sialidases. We show that EstA mediates cross-species sialic acid feeding, driving expansion of sialic acid scavengers. Although the current study is limited to *in vitro* experiments, our results are consistent with recent findings in mouse models, which demonstrate that sialidases produced by the gut microbiota can liberate sialic acids, which in turn provide a metabolic boost that drives the expansion of potential pathogens (31), including *E. coli*. Together with these previous studies, the work presented here suggests that EstA could enable an even more robust sialidase-dependent expansion of sialic acid scavenging pathogens by allowing more extensive liberation of sialic acids from the mucosa. Further investigations are warranted to investigate the role of EstA in the intestine.

Previous studies suggest that bacterial *O*-acetylesterases in the intestine may have pathologic roles in ulcerative colitis (UC) (17, 20). Studies of fecal extracts from individuals with UC have demonstrated that these individuals have higher levels of sialic acid *O*-acetyl esterase activity compared with normal controls, although interestingly, sialidase levels were not different between these groups (17, 20). We have noticed that among the *Bacteroides*, some species appear to encode both sialidase and esterase (*e.g. B. fragilis*), whereas others have only sialidase (*e.g. B. thetaiotaomicron*). Thus, the finding of higher *O*-acetylesterase activity without higher sialidase suggests that a greater portion of the sialidase-producing taxa present in the UC microbiota may produce *O*-acetylesterases. Indeed, one recent study cultured biopsy tissue from individuals with UC and supported the idea that there may be different spectrum of *Bacteroides* species in UC (65). Using an experimental approach in mice, overgrowth of *Bacteroidetes*, *Clostridiales*, and *Enterobacteriacea* among other taxa was observed in a mouse model of UC (DSS-induced colitis) (66). Taking this model one step further, it was shown that the overpopulation by certain *Bacteroides* species in the DSS-induced colitis model increased sialidase activity and subsequent sialic acid-mediated outgrowth of *E. coli*, which in turn led to further exacerbation of inflammation and disease (32). Finally, consistent with these studies in mice, the overrepresentation of *Enterobacteriaceae* was associated with increased severity of disease in UC patients receiving fecal transplants (67). Taken together, these findings support the interpretation that sialic acid cross-feeding may be fueling dysbiotic changes in the microbiota that have been linked to UC and further highlight the potential contribution of *Bacteroides* sialic acid *O*-acetylesterases in this process.

Recent findings in the biology of mucin synthesis, secretion, and turnover in the distal colon illustrate a biological system in which two layers of mucus play key roles in biology and disease. The innermost mucus layer is firmly attached (68), turns over rapidly (69), and typically remains free of bacteria in the healthy colon (6). In contrast, a diverse community of microbes lives within the outer mucus layer (70), where they produce enzymes that facilitate the digestion and metabolism of mucin carbohydrate chains (71, 72). Sonnenburg and co-workers (2) have convincingly argued that capabilities in glycan foraging likely evolved as part of the healthy relationship between symbiotic bacteria and the mammalian host. Such a relationship requires mechanisms to limit host-microbe interactions that may lead to inflammatory or infectious disease. Within the inner mucus layer, host proteins such as Muc2, RegIIIγ, and Lydp8 exclude bacteria from the inner mucus layer (73–75).

The data presented here suggest another mechanism by which the carbohydrate chains of the inner mucus layer may be protected from secreted bacterial enzymes in the gut lumen. Specifically, 7-*O*-acetylation of sialic acid contributes to the integrity of glycan chains against EstA. However, upon migration to the 9-carbon position (9-*O*-acetylated sialic acid), the *O*-acetyl ester becomes susceptible to the action of EstA. The fact that EstA permits community foraging only after the *O*-acetyl group has migrated to C-9 suggests the possibility of mucin sialic acid “maturation” that protects mucin glycans immediately after their secretion, when mucins are closest to the epithelium. Given the published rates of spontaneous 7-*O*-acetyl ester migration toward the 9-position (53), EstA-susceptible *O*-acetyl esters at the 9-position would likely be located exclusively in the aged, outermost mucus layer. This interpretation is supported by both the kinetics of *O*-acetyl migration (*t*_½_ of several hours at pH 7.4) (53), as well as the kinetics of turnover of the inner mucus layer (∼1 h) (69). The likelihood that this mechanism contributes to the healthy host-microbiome interaction is further supported by a recent metagenomic study (comparing twins discordant for malnutrition phenotypes), which identified sialic acid *O*-acetylesterase as one of the most significant biomarkers in the healthy maturation of the gut microbiome (76).

Unfortunately, several factors limit our ability to examine the biology of *O*-acetyl migration and its influence on sialic acid foraging by bacterial populations *in vivo*. First, sialic acid *O*-acetyl esters are extremely labile and will not survive most processes of tissue preparation for staining or other analyses. Second, the tools with which to monitor the placement or movement of these glycan modifications *in vivo* are extremely limited. Finally, unlike many other aspects of mammalian biology, the changes in glycan structure under study here cannot be programmed at the genetic level. We reason that attempts to modulate pH *in vivo* (analogous to what we have done for the *in vitro* biochemical work shown here) would no doubt create many pleiotropic changes in the system and thus would limit the interpretation of the work. It may be more straightforward (though far from trivial) to follow up on the current biochemical experiments using genetic systems in *Bacteroides* together with complex co-colonization experiments in gnotobiotic mice. Given the prior studies in humans that implicate EstA or sialylate *O*-acetyl esterase activity in the healthy maturation and dysbiosis of the gut microbiome, such work is warranted to help evaluate whether the biochemical mechanism of EstA described here may be acting *in vivo* to drive bacterial colonization and/or disease.

## Experimental procedures

### Bacterial strains, plasmids, and culture conditions

Strains and plasmids used in this study are described in Table 1. LB, Circlegrow broth (MP Biomedicals), Todd Hewitt broth (IBI Scientific), and M63 (without glucose or glycerol) were used as bacterial growth media. For plasmid maintenance, antibiotics were used at the following concentrations: ampicillin (Sigma), 100 μg/ml; and erythromycin (Sigma), 5 μg/ml.

**Table 1:** **Strains and plasmids used in this study**

Strain or plasmid	Relevant characteristics	Source/reference
*E. coli* MG1655	Wild-type K12 strain	Coli Genetic Stock Center
*E. coli* EDL933	Wild-type 0157:H7 strain	Gift from Chia Hung
*E. coli* BL21 (DE3)	Expression strain	New England Biolabs
*E. coli* C600	Expression strain	Gift from Scott Hultgren
*E. coli* LSR4	MG1655 Δ*nanA* sialate lyase mutant	This study
*B. fragilis* NCTC 9343	Wild-type strain	DNA from this strain was a gift from Jeffrey Gordon
*B. thetaiotaomicron* VPI-5482	Wild-type strain	DNA from this strain was a gift from Jeffrey Gordon
*S. agalactiae* COH1 Δ*neuA* p*neuA* N301A	Expresses high levels of 7-*O*-acetylated sialic acid	Ref. 23
pET101/d-Topo	Expression vector	Invitrogen
pTrc99A	Expression vector	Gift from Scott Hultgren
pLR6	*B. fragilis nanH his* in pET101/D	This study
pES1	*B. thetaiotaomicron nanH his* in pET101/D	This study
pLR8	*B. fragilis estA his* in pTrc99A	This study

### Accession numbers

This paper performed genetic and/or biochemical characterizations relevant to the following accession numbers: *B. fragilis* NanH sialidase (WP_010992682), *B. thetaiotaomicron* NanH sialidase (WP_008766031), *B. fragilis* EstA (WP_005794991), and *E. coli* NanA sialic acid lyase (WP_000224714).

### DNA manipulations

Primers (Integrated DNA Technologies) used in this study are shown in Table 2. PCR products for cloning were generated with Phusion polymerase (New England Biolabs). Restriction endonucleases were also from NEB. The *nanH* genes from *B. fragilis* and *B. thetaiotaomicron* were amplified with the primer sets *B. fragilis* nanH F, *B. fragilis* nanH R, *B. thetaiotaomicron* nanH F, and *B. thetaiotaomicron* nanH R. PCR products were desalted and cloned into pET101/d-Topo (Invitrogen) according to the manufacturer's instructions. *B. fragilis estA* was amplified with *B. fragilis* estA F Nco and *B. fragilis* estA his R Bam. The amplicon was desalted, digested with NcoI and BamHI, and cloned into the NcoI and BamHI sites of pTrc99A. The Δ*nanA* mutation in MG1655 was made using the phage lambda Red recombinase method (77).

**Table 2:** **Primer sequences**

Primer name	Sequence
*B. fragilis* nanH F	CACCATGAAAAAAGCCGTAATTC
*B. fragilis* nanH R	TTTAATAATGTCTTTCAAC
*B. thetaiotaomicron* nanH F	CACCATGAAAAGAAATCATTATTTATTTACCC
*B. thetaiotaomicron* nanH R	TCGAATCAAATCTTTCAGTTTTAC
*B. fragilis* EstA F Nco	AAAACCATGGCAAAGAAAATTTTATTAATGATGTTGTTGCTC
*B. fragilis* EstA his R Bam	TTTTGGATCCTTAGTGGTGGTGGTGGTGGTGTTTCTTATTTATATAAGGTTTAACAATATCAACC
nanA KO F	ATAAAGGTATATCGTTTATCAGACAAGCATCACTTCAGAGGTGTAGGCTGGAGCTGCTTC
nanA KO R	TTCCCCTCACCCGGTAGGGGCGAGCGAGGGGAAACAACTCATTCCGGGGATCCGTCGACC

### Expression and purification of B. fragilis NanH-his and B. thetaiotaomicron NanH-his

*E. coli* BL21(DE3) containing pES1 (*B. thetaiotaomicron nanH-his* in pET101/D) or pLR6 (*B. fragilis nanH-his* in pET101/D) were grown shaking in 500 ml of Circlegrow broth at 37 °C to an *A*_600_ of ∼1.0. Cultures were induced with 300 μm isopropyl β-d-thiogalactopyranoside (Gold Biotechnology) and grown shaking overnight at room temperature. Bacteria were pelleted at 12,000 × *g*, and the supernatant was passed through a 0.22-μm filter to remove any remaining cells. Then 2 ml of His-Select nickel affinity gel slurry (Sigma) was added to the supernatant and rotated at 4 °C for 90 min. The supernatant was transferred to a glass column with a stopcock, and the beads were allowed to settle over the filter. Once the supernatant was drained, the beads were washed with 200 ml of wash buffer (50 mm NaH_2_PO_4_, pH 7.4, 300 mm NaCl, 20 mm imidazole, chemicals obtained from Sigma). Sialidases were eluted in eight 150-μl fractions of imidazole elution buffer (50 mm NaH_2_PO_4_, pH 7.4, 300 mm NaCl, 250 mm imidazole). Fractions were tested for activity with the fluorogenic sialidase substrate 4-methylumbelliferyl-*N*-acetylneuraminic acid (4-MU-Sia; Gold Bio). In a black polypropylene assay plate (Eppendorf), 10 μl of each fraction was mixed with 100 μl of PBS, pH 7.4, containing 100 μm 4-MU-Sia. 4-Methylumbelliferone fluorescence was measured at excitation of 365 nm (bandwidth, 9 nm) and emission of 440 nm (bandwidth, 20 nm) every 60 s in a Tecan Infinite M200 plate reader.

### Expression and purification of B. fragilis EstA

*E. coli* C600 containing pLR8 (*B. fragilis estA-his* in pTrc99A) or pTrc99A (vector control) were grown shaking in 750 ml of Circlegrow broth at 37 °C to an *A*_600_ of ∼1.0 before being induced with 100 μm isopropyl β-d-thiogalactopyranoside. Cultures were moved to room temperature and allowed to grow overnight. The next day, bacteria were pelleted at 12,000 × *g*, washed once in 250 ml of PBS, and resuspended in 40 ml of 50 mm Tris, pH 8.0, 10 mm EDTA, and 20% sucrose. Lysozyme (Sigma) was added to 1 mg/ml, and the suspensions were incubated on ice for 45 min. Bacteria were pelleted, and the supernatants (containing the periplasm fraction) were transferred to 25-cm lengths of 8-kDa cutoff dialysis tubing (BioDesign). Supernatants were dialyzed twice for 1 h at 4 °C against 1 liter of lysis buffer (50 mm NaH_2_PO_4_, pH 7.4, 300 mm NaCl, 10 mm imidazole), and a final time overnight at 4 °C against 1 liter of lysis buffer. The dialyzed material was transferred to 50-ml Falcon tubes containing 700 μl of His-Select nickel affinity gel slurry, rotated for 2 h at 4 °C, then applied to 5-ml disposable polypropylene columns (Thermo Scientific), and washed with 10 ml of wash buffer (see above). Bound proteins were then eluted in eight 250-μl fractions of imidazole elution buffer.

Purity of the eluted EstA-His_6_ (and control material prepared in parallel from cells containing empty vector) was evaluated by SDS-PAGE (on a 4–20% gradient gel) followed by staining with Coomassie G-250 (Bio-Rad). This revealed a single prominent band of the expected molecular mass (23 kDa) with the highest concentration in fraction 4 (the fraction used in our enzyme assays; Fig. 2*B*). There were no evident bands in lanes loaded with empty vector control elutions. To further verify that this band corresponded to purified EstA, we ran a second SDS-PAGE gel and transferred the proteins to nitrocellulose for Western blotting with anti-His_6_ monoclonal antibody (Covance). A single band at 23 kDa was recognized by the antibody in lanes loaded with EstA-His_6_, but not those loaded with vector control elutions, indicating that the purified protein is indeed EstA-His_6_ (Fig. 2*D*). Protein concentration was determined with a micro-BCA protein assay kit (Thermo Scientific).

### Sialic acid measurements

Derivatization and quantitation of sialic acids by HPLC was carried out as previously described (56). For measurement of total sialic acids (including bound and free), mild acetic acid hydrolysis of samples (2 m acetic acid for 3 h at 80 °C) or removal of sialic acids using AUS was performed prior to derivatization with 1,2,-diamino 4,5-methylenedioxybenzene dihydrochloride (DMB) as described below. Alternatively, when the experimental question was the extent of sialic acid liberation under different conditions, free sialic acid levels were measured by DMB derivatization without prior acid hydrolysis or treatment with AUS. The samples were mixed with 2× DMB reagent consisting of 14 mm DMB, 44 mm sodium hydrosulfite, 1.5 m 2-mercaptoethanol, and 2.8 m acetic acid and incubated at 50 °C for 2 h. Derivatized samples were injected into a Waters HPLC equipped with a reverse-phase C18 column (Tosoh Bioscience) and a Waters fluorescence detector set to excite at 373 nm and detect emission at 448 nm. Peak integrations were used to quantitate sialic acid content by referencing a standard curve of sialic acid (Neu5Ac; Sigma) derivatized in parallel.

### Sialic acid consumption assays

*E. coli* strains MG1655 (commensal isolate) and EDL933 (enterohemorrhagic isolate) were grown shaking in LB at 37 °C overnight. The next day, 1 μl of each stationary phase culture was inoculated into 500 μl of fresh LB. Sterile filtered Neu5Ac (Sigma) was added to a final concentration of 100 μm where indicated. For Fig. 1*C*, samples were prepared similarly, but 5 μl of purified *B. fragilis* NanH, *B. thetaiotaomicron* NanH, or commercially available *V. cholerae* sialidase (Sigma) was added to 495 μl of LB prior to inoculation with *E. coli*. Bacteria were grown overnight as above. The following day, 100 μl of each culture was transferred to a fresh tube for acid hydrolysis and quantification of total sialic acid by DMB HPLC. The remaining cultures were spun at 16,000 × *g* for 2 min, and supernatants were removed for quantification of free sialic acid by DMB-HPLC.

### Chemical de-O-acetylation of BSM sialic acids

*O*-Acetyl groups were chemically removed from BSM sialic acids by treating samples with 100 mm NaOH for 30 min at 37 °C. Samples were then neutralized with glacial acetic acid. Mock samples received a salt solution containing equivalent amounts of NaOH and acetic acid used for chemical de-*O*-acetylation (base and acid were combined before being added to each mock-treated sample). For the experiment presented in Fig. 2*A*, base treatment was performed prior to enzyme reactions to determine how *O*-acetylation influenced the ability of sialidases to liberate BSM sialic acids. To allow accurate comparison of mock-treated samples with those that were initially de-*O*-acetylated, all samples were base-treated following reactions with *Bacteroides* sialidases.

### Sialic acid release from chemically de-O-acetylated or mock-treated BSM

Bovine submaxillary mucin (Sigma) was dissolved in double-distilled water at 10 mg/ml, and insoluble debris was removed by centrifugation at 16,000 × *g*. Following base treatment (described above), base and mock-treated samples were mixed with NaOAc, pH 5.5, to a final BSM concentration of 2 mg/ml and a final buffer concentration of 20 mm. Purified *Bacteroides* sialidases were added at ∼50 milliunits/ml, which was estimated using our positive control enzyme, AUS (EY Laboratories) using 4-MU-Sia as substrate as described above. The samples were incubated with enzymes at 37 °C for 5 h, followed by NaOH treatment (and neutralization, as described above), DMB derivatization, and HPLC. The second base treatment was performed to allow comparison of the Neu5Ac and Neu5Gc peaks between samples that were initially base *versus* mock-treated.

### EstA O-acetylesterase assay using bovine submaxillary mucin as substrate

Bovine submaxillary mucin (Sigma) was dissolved in double-distilled water at 10 mg/ml, and insoluble debris was removed by centrifugation at 16,000 × *g*. For the experiment presented in Fig. 2*C*, mucin was diluted to 2 mg/ml in PBS (pH 7.4) and mixed with a final concentration of 20 μg/ml purified EstA-His or an equivalent amount of the vector control elution (both added at a 1/20 dilution). The samples were incubated at 37 °C for 2 h. Bound sialic acids were then released with 250 millunits/ml AUS for 1 h at 37 °C, and DMB-HPLC was performed. In this experiment, AUS was used in excess to enzymatically release sialic acids quickly and to avoid using acetic acid hydrolysis of sialic acids, which itself causes some migration and loss of *O*-acetyl groups (and therefore interferes with the interpretation of the experiment).

### EstA-assisted release of BSM sialic acids by Bacteroides sialidases

BSM was dissolved in water, centrifuged as above, and then diluted to 2 mg/ml in PBS, pH 7.4, or 100 mm NaOAc, pH 5.5. The BSM preparation was mixed with purified EstA-His at a final concentration of 20 μg/ml (or an equivalent amount of control elution), and with purified *B. fragilis* or *B. thetaiotaomicron* NanH-His, added at ∼50 milliunits/ml (based on estimations in comparison to AUS using 4-MU-Sia as substrate). Following incubation at 37 °C for 5 h, samples were base-treated as described above to chemically remove *O*-acetyl esters prior to DMB derivatization and HPLC, allowing an accurate quantitation of sialic acids (Fig. 2, *E–H*).

### E. coli growth on mucin in the presence of EstA and/or sialidases

BSM was dissolved in M63 medium, pH 7.0, at 15 mg/ml. Insoluble material was removed by centrifugation at 16,000 × *g*, and the supernatant was passed through a sterile 0.45-μm filter. 150 μl of the soluble, filter sterilized BSM preparation was mixed with 1.5 μl of EstA (for a final concentration of 4 μg/ml) or control elution and 1.5 μl of purified *B. fragilis* or *B. thetaiotaomicron* NanH (∼10 milliunits/ml estimated as described above). 0.5 μl of MG1655 or LSR4 (MG1655 Δ*nanA*) overnight culture grown in LB was added, and the mixtures were transferred to a Greiner sterile clear flat-bottomed 96-well plate. The plate was then tightly covered with a transparent seal (TempPlate RT USA Scientific), and *A*_600_ was measured every 30 min for 22 h at 37 °C in a Tecan M200 plate reader. Before each optical density measurement, the plate was shaken for 20 s to ensure that the bacteria were resuspended.

### EstA O-acetylesterase assay using sialic acids from Group B Streptococcus as substrate

*Streptococcus agalactiae*, also known as GBS was used as a model system for defining the preferences of *O*-acetyl placement and migration on EstA activity. Strain COH1 Δ*neuA* expressing *neuA* N301A from a plasmid was used because it bears a mutated inactive version of its own sialic acid *O*-acetyl esterase, which normally removes *O*-acetyl esters of free sialic acids intracellularly, prior to capsule synthesis (23). The result is that the NeuA N301A strain expresses high levels of sialic acid *O*-acetylation that begins at C-7 and can be migrated to the 9-carbon position. Bacteria were grown to *A*_600_ = 0.4 in 20 ml of Todd Hewitt broth with 5 μg/ml erythromycin to maintain the *neuA* N301A plasmid. The culture was divided into two Oakridge tubes and centrifuged at 12,000 × *g* for 10 min. The cells were washed three times in 100 mm NaOAc, pH 5.5. One pellet was resuspended in 100 mm Tris, pH 9.0, and incubated at 37 °C for 30 min to migrate *O*-acetyl groups to the 9-carbon position. The other pellet was resuspended in 210 μl of PBS, pH 7.4, and incubated on ice. The cells in Tris were pelleted and washed once in 1 ml of PBS and then resuspended in 210 μl of PBS. 30 μl of each suspension was transferred to fresh tubes for 0′ time points. 90-μl aliquots of the remaining suspensions were mixed either with 10 μl of purified EstA (for a final concentration of 40 μg/ml) or an equivalent volume of vector control elution. The four mixes were then divided into three tubes each of 30 μl and incubated for 30, 90, or 300 min at 37 °C. At each time point, the cells were pelleted and washed with 1 ml of 100 mm NaOAc, pH 5.5. Bacteria were resuspended in 30 μl of 20 mm NaOAc, pH 5.5, containing 250 milliunits/ml AUS and incubated for 1 h at 37 °C. The cells were pelleted, and 20 μl of supernatant was removed and diluted 4× in double-distilled water prior to DMB derivatization and HPLC.

## Author contributions

A. L. L. and W. G. L. conceived the idea for the project. L. S. R. performed most of the experiments and wrote first drafts of the “Results” and “Experimental procedures.” All authors participated in experimental design and data analysis/interpretation. A. L. L. wrote first drafts of the introduction and “Discussion” and referenced the manuscript. All authors edited the manuscript and approved it in its current form.

## Footnotes

3 The abbreviations used are:

Neu5Ac: *N*-acetylneuraminic acid
DSS: dextran sulfate sodium
LB: lysogeny broth
BSM: bovine submaxillary mucin
AUS: *A. ureafaciens* sialidase
Neu5Gc: *N*-glycolylneuraminic acid
GBS: group B *Streptococcus*
UC: ulcerative colitis
DMB: 1,2,-diamino 4,5-methylenedioxybenzene dihydrochloride
4-MU-Sia: 4-methylumbelliferyl-*N*-acetylneuraminic acid
ANOVA: analysis of variance.
